# Femicides in Turkey: Analysis of the last two decades’ cases

**DOI:** 10.1093/eurpub/ckac129.617

**Published:** 2022-10-25

**Authors:** NP Erbaydar, ZD Muharremoğlu, DA Mutlu

**Affiliations:** 1 Public Health Department, Hacettepe University Medical Faculty, Ankara, Turkey; 2 Public Health Service Headship, Province Health Directorate, Sakarya, Turkey; 3 Department of Political Economy, Atılım University, Institute of Social Science, Ankara, Turkey

## Abstract

**Aim:**

Femicide is the women's murder by men for misogynistic reasons. This research aims to determine the characteristics of femicides in Turkey.

**Methods:**

In this cross-sectional study, 13 news sites were scanned using keywords related to femicides between 2000-2019, and 1744 cases were included. In the study, the murdered women, the perpetrators’, and the femicides’ characteristics were determined and the percentages, central tendency, and distribution measures were calculated.

**Results:**

The mean age of the murdered women was 33.2 (SD = ±13.2); 55.5% who were married or cohabitated had at least one child (Mean=2.2±1.4), and 977 children lost their mothers due to femicide. Of the women killed, 19.1% of them filed for divorce, 10.0% of them applied to law enforcement before, and 4.5% were pregnant. Of the perpetrators, 53.1% were husbands/ex-husbands. The mean age of perpetrators was 38.3±13.4 and 10.8% had criminal records. Of the perpetrators, 12.1% had threatened the women before, and 6.2% of them had been detained. Of the femicides, 80.4% were committed in urban areas. According to the news, 34.7% of the perpetrators were in the prosecution and detention process, 25.6% were on trial, 16.2% committed suicide. Of those whose sentences have been finalized, 26.6% of them were given aggravated life imprisonment and 36.3% of them to life imprisonment. Sudden quarrels, desire for divorce, jealousy, and honor/custom were the most cited reasons. Firearms or sharps were the most frequently used weapons.

**Conclusions:**

Women were killed by men who were close to them. Women's desires for separation/divorce or to make their own decisions for their lives posed a threat to traditional masculinity. The fact that one out of every ten perpetrators had a criminal record in femicides showed that violence was a way of life for some men. In addition, the ease of access to firearms led to their frequent use in femicides.

**Key messages:**

• Improve gender equality.

• Access to firearms should prevented.

